# Emotion recognition of morphed facial expressions in presymptomatic and symptomatic frontotemporal dementia, and Alzheimer’s dementia

**DOI:** 10.1007/s00415-020-10096-y

**Published:** 2020-07-29

**Authors:** Lize C. Jiskoot, Jackie M. Poos, Manon E. Vollebergh, Sanne Franzen, Judy van Hemmen, Janne M. Papma, John C. van Swieten, Roy P. C. Kessels, Esther van den Berg

**Affiliations:** 1 Department of Neurology, Erasmus Medical Center, NF-331, Post box 2040, 3000 CA Rotterdam, The Netherlands; 2 Department of Radiology, Leiden University Medical Center, Leiden, The Netherlands; 3 Dementia Research Centre, University College London, London, UK; 4 Donders Institute for Brain, Cognition and Behaviour, Radboud University Nijmegen, Nijmegen, The Netherlands; 5 Department of Medical Psychology, Radboudumc Alzheimer Center, Nijmegen, The Netherlands

**Keywords:** Frontotemporal dementia, Alzheimer’s dementia, Emotion recognition, ERT, Presymptomatic, Familial

## Abstract

**Background:**

The emotion recognition task (ERT) was developed to overcome shortcomings of static emotion recognition paradigms, by identifying more subtle deficits in emotion recognition across different intensity levels. In this study, we used the ERT to investigate emotion recognition deficits across the frontotemporal (FTD) and Alzheimer’s Dementia (AD) spectrum.

**Methods:**

With the ERT, we assessed the recognition of facial emotional expressions (anger-disgust-fear-happiness-sadness-surprise) across four intensities (40–60–80–100%) in patients with behavioural variant FTD (bvFTD; *n* = 32), and AD (*n* = 32), presymptomatic FTD mutation carriers (*n* = 47) and controls (*n* = 49). We examined group differences using multilevel linear regression with age, sex and education level as covariates, and performed post hoc analyses on presymptomatic (*MAPT*, *GRN* and *C9orf72*) mutation carriers. Classification abilities were investigated by means of logistic regression.

**Results:**

Lowest ERT total scores were found in patients with bvFTD and AD, whereas equal highest performance was found in presymptomatic mutation carriers and controls. For all emotions, significantly lower subscores were found in patients with bvFTD than in presymptomatic mutation carriers and in controls (highest *p* value = 0.025). Patients with bvFTD performed lower than patients with AD on anger (*p* = 0.005) and a trend towards significance was found for a lower performance on happiness (*p* = 0.065). Task performance increased with higher emotional intensity, and classification was better at the lowest than at the highest intensity. *C9orf72* mutation carriers performed worse on recognizing anger at the lowest intensity than *GRN* mutation carriers (*p* = 0.047) and controls (*p* = 0.038). The ERT differentiated between patients with bvFTD and controls, and between patients with AD and controls (both *p* < 0.001).

**Discussion:**

Our results demonstrate emotion recognition deficits in both bvFTD and AD, and suggest the presence of subtle emotion recognition changes in presymptomatic *C9orf72*-FTD. This highlights the importance of incorporating emotion recognition paradigms into standard neuropsychological assessment for early differential diagnosis, and as clinical endpoints in upcoming therapeutic trials.

**Electronic supplementary material:**

The online version of this article (10.1007/s00415-020-10096-y) contains supplementary material, which is available to authorized users.

## Introduction

Frontotemporal dementia (FTD) and Alzheimer’s Dementia (AD) are the two most prevalent early-onset types of dementia. The clinical profile of FTD is typically characterized by behavioural and language disturbances, with cognitive deficits in executive function and relative sparing of memory and visuospatial abilities [1, 2], whereas the first symptoms of AD are usually episodic memory and visuospatial impairments [3]. Differential diagnosis in a young-onset population is complicated by frequent atypical presentations and clinical overlap between the two entities, with significant memory deficits in FTD [4], and predominant ‘frontal’ (dysexecutive and behavioural) and language variants of AD being described [5], often leading to misdiagnosis and/or diagnostic delay. Early diagnosis is, however, essential for proper patient and caregiver management and planning, non-pharmacological symptomatic treatment, and patient stratification in upcoming clinical trials [6].

As marked behavioural and emotional changes may already occur in the early disease stages of both FTD and AD, an increasing number of studies emphasize the importance of social-cognitive assessments to improve early diagnosis [7]. Social cognition refers to a broad and complex cognitive concept encompassing the psychobiological processes needed to comprehend and socially interact with other people, often conceptualized along three hierarchical levels, ranging from perception and automatic attribution (e.g., emotion recognition), understanding and interpretation of social information, to reasoning and regulation [8]. Recent meta-analyses have shown consistent deficits across all three levels of social cognition in FTD [7] and mild cognitive impairment (MCI) [9], often considered to be the prodromal phase of AD. Special emphasis is often put on deficits in facial emotion recognition, as they are thought to lie at the base of social cue misinterpretation leading to difficulties with social conduct [10]. Meta-analyses of emotion recognition abilities have shown significant deficits in behavioural (bvFTD,[10] and language variants of FTD (primary progressive aphasia, or PPA [7], as well as MCI [9] and AD [10], but with large variability across studies depending on the specific tasks used. Prodromal FTD studies are lacking thus far, with only one study showing subtle decline over time in presymptomatic FTD mutation carriers [11].

The question is whether traditional measures of social cognition are able to identify the subtle and slowly emerging deficits in the earliest stages of dementia. The Ekman 60 Faces test [12], one of the most often used paradigms, for instance employs static photographs of actors mimicking full-blown emotions. More subtle emotion recognition deficits can, therefore, be missed, as full-blown emotions often do not resemble facial expression in everyday communication. Static images also take natural movement and dynamic development of facial expressions less into account. Moreover, (near) ceiling effects for the emotion happiness are often found, as happy faces are generally more easily recognised in the absence of other positive emotions as possible distractors. This could reduce the test’s sensitivity (i.e. the proportion of patients identified as being impaired), hampering its use in clinical practice [13].

To overcome the shortcomings of the Ekman Faces, the emotion recognition task (ERT) [13, 14] was developed. It presents dynamically morphed facial expressions of the same six basis emotions (happiness, anger, disgust, surprise, sadness and fear), but across different levels of intensity. In that way, the ERT might be more sensitive to detect subtle deficits in the early stages of dementia than the static images used in the Ekman Faces Test. The ERT has been validated in a wide range of neurological diseases, including Huntington’s disease [15], multiple sclerosis [16], traumatic brain injury [17], stroke [18], Korsakoff’s syndrome [19], and Parkinson’s disease [20]. With respect to research into the ERT in the dementia field, a study in a small convenience sample of bvFTD patients demonstrated specific impairments in the recognition of the emotions anger and surprise [14], however, no studies have been performed in presymptomatic FTD yet. The ERT has only been used in one study on MCI and AD [21], but no direct comparisons with bvFTD have been made so far. The aim of the present study is, therefore, to investigate emotion recognition deficits across the different emotions and emotional intensities as well as classification abilities of the ERT in patients with bvFTD and compare them to patients with AD, presymptomatic FTD mutation carriers, and cognitively unimpaired controls, that can be used to improve early differential diagnosis in dementia.

## Methods

### Participants

In this retrospective study, we included data from 32 patients with bvFTD via the outpatient memory clinics of the Erasmus Medical Center (*n* = 22) and Radboud University Medical Center (*n* = 10), the Netherlands. Six bvFTD patients were carrying a pathogenic FTD mutation (chromosome 9 open reading frame 72 repeat expansion (*C9orf72*), all other patients were sporadic. Five other bvFTD patients had concomitant amyotrophic lateral sclerosis (bvFTD-ALS). We included data from 32 patients with AD, who were either assessed at the outpatient memory clinics of the Erasmus Medical Center (*n* = 3) or participated in a previous study for which they were recruited via the outpatient memory clinic of the Zorg Groep Twente (ZGT) hospital in Almelo and Hengelo (*n* = 29), the Netherlands [21]. Diagnoses were made in a multidisciplinary consensus meeting, using established diagnostic criteria for probable bvFTD (*n* = 28) and bvFTD with definite FTLD pathology (*n* = 4) [1], ALS [22], and probable AD [23]. Furthermore, we enrolled 101 participants of the FTD Risk Cohort (FTD-RisC) from the Erasmus Medical Center, in which first-degree family members patients with FTD due to a pathogenic mutation are followed longitudinally [24]. DNA genotyping assigned these participants to either the mutation carrier (*n* = 47) or non-carrier group (controls; *n* = 49). Mutation carriers were from either microtubule-associated protein tau (*MAPT*; *n* = 7), progranulin (*GRN*; *n* = 22) or *C9orf72* (*n* = 18) families. Mutation carriers were deemed to be presymptomatic when they did not fulfill clinical diagnostic criteria for bvFTD [1], PPA [2] or FTD-ALS [22], and had CDR^®^ plus Behaviour and Language domains from the NACC FTLD Module (CDR^®^ plus NACC FTLD) [25] of 0. The investigators and participants were blinded for the genetic status of at-risk participants, except for those that underwent predictive testing at their own request.

All patients with dementia from the outpatient clinic of the Erasmus Medical Center were part of a local biobank study, for which they provided written informed consent for the use of their anonymized medical and clinical data for research purposes. Participants of the FTD-RisC study provided written informed consent for the use of their anonymized research data. The data from the Radboud University Medical Center were collected as part of routine neuropsychological assessments, and stored and analyzed in anonymized form in accordance with the General Data Protection Regulation. Patients provided written informed consent concerning their storage and use. The data from the ZGT hospital were collected as part of another study [21], for which written informed consent was obtained in all patients according to the declaration of Helsinki and the Institutional Review Board of the ZGT hospital gave approval. The Erasmus Medical Center ethics committee gave approval for both the local biobank and the FTD-RisC study.

### Procedure

The ERT was administered as part of the neuropsychological assessment performed during the memory clinic work-up (patients) or study visit (FTD-RisC participants). The Mini-Mental State Examination (MMSE) [26] was administered as measure of global cognitive functioning. The clinical dementia rating scale (CDR) [27] was used as a measure of disease severity in patients with AD, while patients with bvFTD from the Erasmus Medical Center as well as FTD-RisC participants were assessed about functional changes in behaviour, neuropsychiatric symptoms, cognition and language by means of the CDR^®^ plus NACC FTLD [25] during the study visit or afterwards in a telephone interview.

### Emotion recognition task (ERT)

**Fig. 1:**
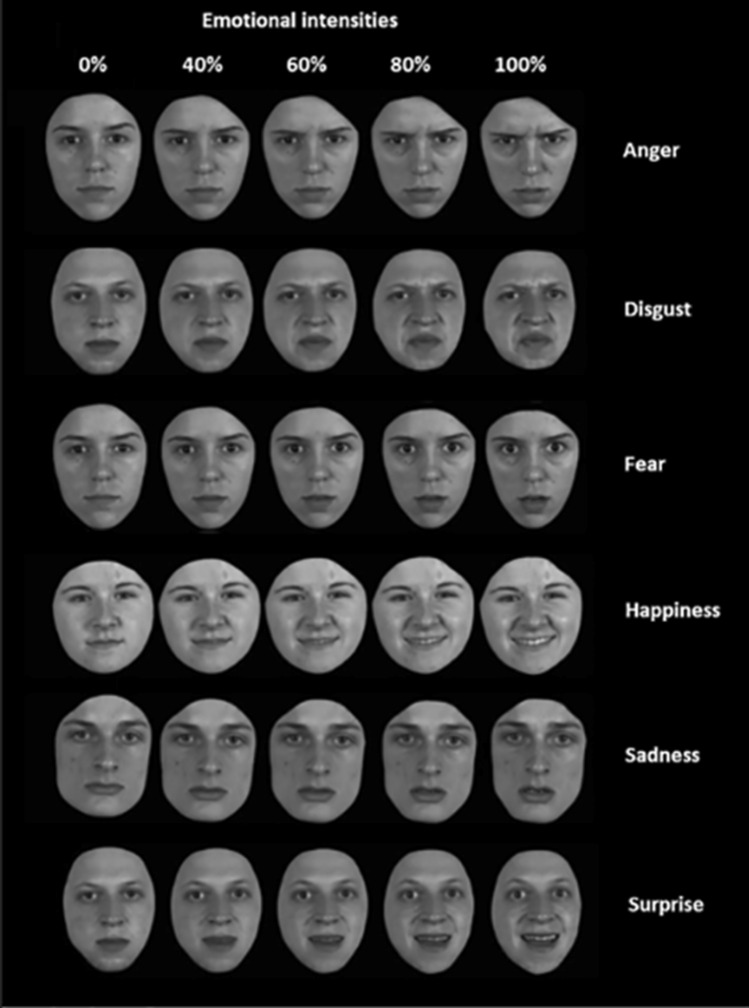
The Emotion Recognition Task. Displayed are examples of facial expressions of six universal emotions (anger, disgust, fear, happiness, sadness, and surprise). The ERT is a computerized test, that enables a real-time interactive morphing between two endpoint facial expressions (0%, i.e. neutral, and 40, 60, 80 or 100% intensity). Adapted with permission from Kessels et al. [13]

Emotion recognition abilities were assessed with the ERT. The ERT is a computerized neuropsychological test, available via the DiagnoseIS neuropsychological assessment system (www.diagnoseis.com). It enables a real-time interactive morphing between two endpoint facial expressions (0% = neutral, and 100% = full − blown emotion) [13, 14]. Each morph was created from 21 images between 0 and 100% intensity, generating video clips in which the degree of emotional expression was increased by 20% steps, starting at 40% intensity. The video clips were presented starting at the lowest intensities (i.e., neutral morphed into 40% intensity to neutral morphed into 100% intensity–see Fig. 1). The duration of the video clips was one (40% intensity) to three (100% intensity) seconds. The ERT starts with a screen presenting the task instructions to the participant in her/his native language. Simultaneously, the examiner reads these instructions aloud to the participant, thereby ensuring minimal variation in the administration procedure. Following the instructions, three practice stimuli are presented, showing respectively an angry, a happy, and a disgusted expression that were not part of the final test set. The instructions and practice trials were repeated if the participant did not understand the instructions. Responses were made by mouse click. If participants were unsure how to or unable to operate the computer mouse, the examiner assisted by asking which label he or she deemed the most appropriate (and clicked the given response if needed). The test was discontinued in case the participant still did not understand the test instructions or did not know how to respond after repeating the instructions. In the real test, all emotions of the same emotional intensity were presented in pseudo-random but fixed order to control for possible order effects of previously encountered emotions. In each trial, the participants had to label the facial emotional expression using a six-alternative forced choice (i.e., anger, disgust, fear, happiness, sadness and surprise). Performance was calculated as the number of correctly labelled expressions per emotion and intensity (maximum = 4). Across the 4 intensities, the maximum score of each emotion was 16, to a total of 96 for the entire test. Administration time was approximately 10 min.

### Statistical analysis

We performed statistical analyses using SPSS Statistics 25.0 (IBM Corp., Armonk, NY). Alpha was set at 0.05 across all comparisons, unless otherwise specified, and two-tailed analyses were performed. We compared continuous demographic data between groups by means of one-way ANOVA for normally distributed data, or Kruskal–Wallis tests in case of non-normally distributed data. We performed post hoc comparisons with Bonferroni (parametric data) or Mann–Whitney *U* (nonparametric data) tests. Between-group differences in sex distribution were analysed using Pearson *χ*^2^ tests. We examined group differences in ERT total and emotion subscores using by means of one-way ANCOVA for normally distributed data, or Quade’s rank analysis of covariance for non-normally distributed data—using age, sex and education level as covariates. To investigate differences between emotions across emotional intensities we used multilevel linear regression modeling, with group as between-subject variable and emotion and emotional intensity as within-subject variables—using raw scores for normally distributed data and, in case of non-normally distributed data, using rank-transformed data. Again, analyses were corrected for age, sex and education level. In post hoc analyses, we explored differences between patients with sporadic bvFTD, *C9orf72*-associated bvFTD, and patients with concomitant ALS, as well as between pathogenic mutations amongst presymptomatic mutation carriers (*MAPT*, *GRN* and *C9orf72*) and controls. We performed multinomial logistic regression analyses, and determined sensitivity and specificity by the area under the curve (AUC) by receiver operating characteristic (ROC) analyses to investigate the classification abilities of the ERT between the subgroups. We first checked for non-linearity, dependence of errors and multicollinearity. All analyses were adjusted for age, sex and education level. Optimal cut-off levels were given by the highest Youden’s index [28]. The models were selected with a forward stepwise method according to the likelihood ratio test and applying the standard *p* values for variable inclusion (0.05) and exclusion (0.10). Goodness of fit was evaluated with the HL *Χ*^2^ test. Nagelkerke *R*^2^ is reported as measure of effect size. To correct for the potential influence of our data coming from different cohorts, we reran all analyses using centre as a covariate.

## Results

### Demographics data

Demographic and clinical data of patients with bvFTD and AD, presymptomatic mutation carriers, and controls are shown in Table 1. Patients with AD were significantly older than patients with bvFTD (*U* = 135.5, *p* < 0.001), presymptomatic mutation carriers (*U* = 29, *p* < 0.001) and controls (*U* = 61, *p* < 0.001), and patients with bvFTD were significantly older than presymptomatic mutation carriers (*U* = 278, *p* < 0.001) and controls (*U* = 421, *p* < 0.001). The patients with AD had a lower education level than mutation carriers and controls (*p* < 0.001), and patients with bvFTD (*p* = 0.039). MMSE scores were highest in the presymptomatic mutation carriers and controls, being significantly higher than in patients with bvFTD (bvFTD vs. presymptomatic mutation carriers: *U* = 145.5, *p* < 0.001; bvFTD vs. controls: *U* = 179, *p* < 0.001). MMSE scores were lower in patients with AD than in all other subgroups (AD vs. bvFTD: *U* = 146.5, *p* < 0.001; AD vs. presymptomatic mutation carriers: *U* = 14, *p* < 0.001; AD vs. controls: *U* = 19, *p* < 0.001). There were no significant differences in sex between groups (*Χ*(4) = 3.08, *p* = 0.38). Disease duration (*U* = 44, *p* = 0.85) and stage (CDR^®^/CDR^®^ plus NACC FTLD scores) did not differ between patients with bvFTD and AD (Table 1). There were no significant differences regarding demographic or clinical data between the presymptomatic mutation carriers and controls. There was, however, a significant age difference between presymptomatic mutation carrier groups [*H*(2) = 7.31, *p* < 0.026], with *C9orf72* mutation carriers being younger than *GRN* mutation carriers (*U* = 105, *p* = 0.011).

**Table 1:** Demographic and clinical data per subgroup

	bvFTD patients (*n* = 32)	AD patients (*n* = 32)	Presymptomatic mutation carriers (*n* = 47)	Controls (*n* = 49)
Age, year [range]	63.0 ± 9.9 [35.8–79.8]	76.0 ± 6.8 [62.1–87.0]	48.7 ± 12.6 [23.4–76.1]	52.4 ± 13.3 [34.8–74.5]
Female (%)	14 (43.8)	19 (59.4)	29 (61.7)	25 (51.0)
Gene in family	*MAPT* *n* = 0 *GRN* *n* = 0 *C9orf72* *n* = 4	n/a	*MAPT* *n* = 7 *GRN* *n* = 22 *C9orf72* *n* = 18	*MAPT* *n* = 10 *GRN* *n* = 26 *C9orf72* *n* = 13
Education (level)*	4.7 ± 1.3	3.9 ± 1.3	5.5 ± 1.0	5.5 ± 0.9
Disease duration, year [range]	4.3 ± 2.8 [0.7–11.3]	5.2 ± 6.0 [0.7–12.0]	n/a	n/a
MMSE (max. 30)	25.6 ± 4.0	19.4 ± 4.7	29.4 ± 0.8	29.3 ± 0.9
CDR^©^ (plus NACC FTLD), range†	0.5–2.0	1.0–2.0	0	0
ERT total score (max. 96)	42.9 ± 14.3	40.6 ± 9.8	58.6 ± 7.2	51.0 ± 12.4
Anger subscore (max. 16)	8.3 ± 3.8	9.1 ± 3.6	13.6 ± 2.1	13.6 ± 2.4
Disgust subscore (max. 16)	6.9 ± 4.4	5.6 ± 3.4	116 ± 3.0	10.3 ± 3.7
Fear subscore (max. 16)	5.6 ± 5.5	3.0 ± 2.1	3.9 ± 2.9	4.1 ± 3.4
Happiness subscore (max. 16)	9.6 ± 5.6	12.6 ± 2.3	15.0 ± 1.1	15.1 ± 1.2
Sadness subscore (max. 16)	5.6 ± 4.5	3.7 ± 2.6	6.5 ± 3.2	5.8 ± 2.8
Surprise subscore (max. 16)	7.0 ± 4.1	6.5 ± 2.9	8.0 ± 2.6	7.3 ± 2.4

Values indicate mean ± SD or *n* (%)

*bvFTD* behavioural variant frontotemporal dementia, *AD* Alzheimer’s Dementia, *MMSE* Mini-Mental State Examination, *CDR* clinical dementia rating, *NACC* National Alzheimer’s Coordinating Center, *FTLD* frontotemporal lobar degeneration, *ERT* Emotion Recognition Test

*Dutch educational system categorized into levels from 1 = less than 6 years of primary education to 7 = academic schooling [29]

^†^The CDR weighted score was used for patients with AD, whereas the CDR^©^ plus NACC FTLD weighted score was used for patients with bvFTD, presymptomatic mutation carriers and controls; CDR^©^ plus NACC FTLD scores were available for 22/32 bvFTD patients

### Group differences on the ERT

**Fig. 2:**
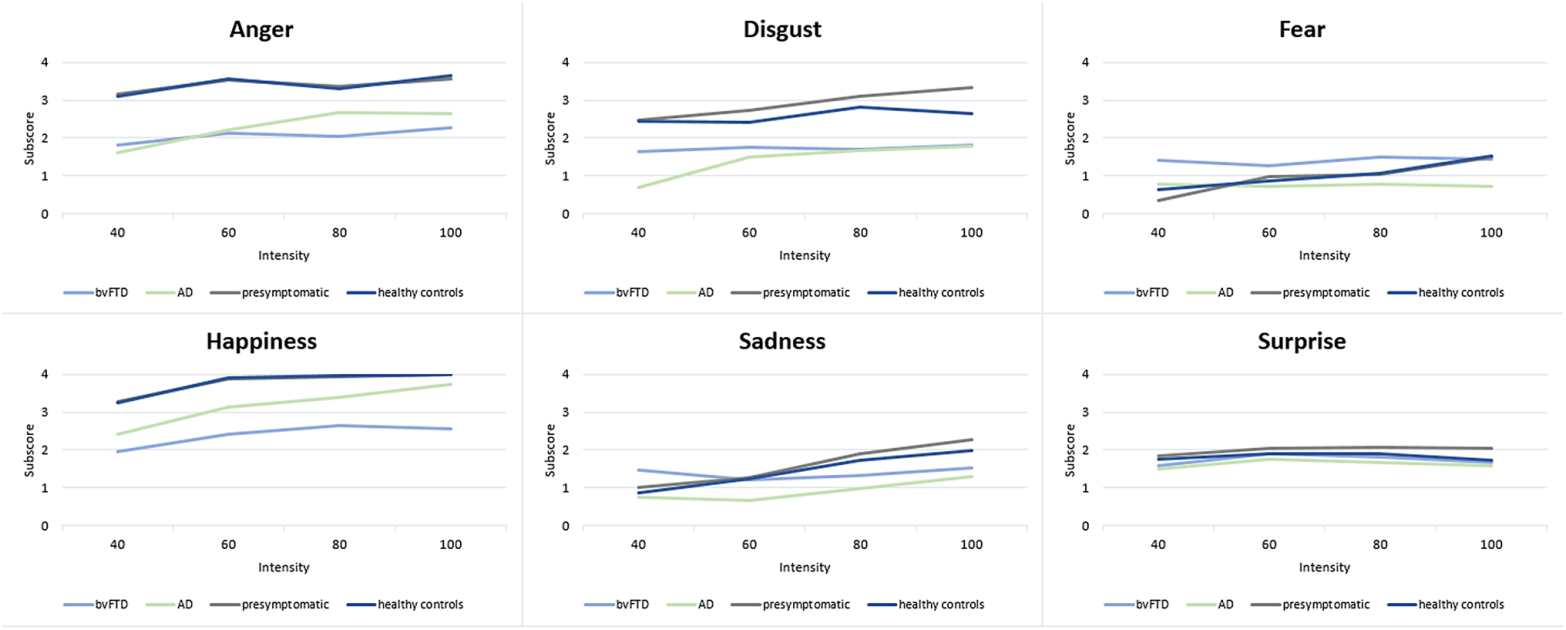
Mean performance (*y*-axis, number correctly identified emotions = max 4) of patients with bvFTD (light blue), patients with AD (light green), presymptomatic mutation carriers (dark grey), and cognitively unimpaired controls (dark blue) for the six different emotions across the emotional intensities (*x*-axis)

As there were no significant differences in total ERT or ERT subscores between sporadic bvFTD patients, bvFTD patients carrying the *C9orf72* mutation, or bvFTD patients with concomitant ALS (see Supplementary Table 1), we pooled the three subtypes into one bvFTD group. There were significant differences in ERT total score between groups [*F*_(3,161)_ = 31.13, *p* < 0.001] (Table 1). Patients with bvFTD had lower scores than patients with AD (*p* = 0.001), presymptomatic mutation carriers (*p* < 0.001) and controls (*p* < 0.001), and also patients with AD had lower ERT total scores than presymptomatic mutation carriers (*p* < 0.001) and controls (*p* < 0.001). There were no significant differences in ERT total scores between presymptomatic mutation carriers and controls (*p* = 0.250). Apart from fear [*F*_Quade(3,145)_ = 1.32, *p* = 0.270], all ERT subscores showed significant differences between groups (*p ≤ *0.011) (Table 1). The lowest scores, regardless of clinical status, were found for the identification of the emotions fear and sadness, followed by surprise and disgust (Fig. 2). Patients with bvFTD performed lower than patients with AD on the emotions anger (*p* = 0.005) and a trend towards significance was found for happiness (*p* = 0.065) (Table 1, Fig. 2). For all emotions, significantly lower subscores were found in patients with bvFTD than in presymptomatic mutation carriers and controls (highest *p* value = 0.025). Patients with AD had lower disgust scores than presymptomatic mutation carriers (*p* = 0.013), but did neither differ regarding other subscores nor from controls. For all emotions, performance was almost identical in the presymptomatic mutation carriers and controls (*p* = 1.00; Fig. 2). All emotions, irrespective of clinical status, showed a similar pattern of increasing performance with higher emotional intensity [*F*_(3,460)_ = 3.80, *p* = 0.01]. Differences between groups were the largest at the lowest intensity (40%) than at the highest intensity (100%) for the emotions disgust (*p* = 0.028), fear (*p* = 0.006), and sadness (*p* = 0.03). Rerunning our analyses using centre as additional covariate did not change aforementioned results.

ERT total scores did not differ between the presymptomatic *MAPT*, *GRN* and *C9orf72* mutation carriers and the controls [*F*_(3,92)_ = 1.19, *p* = 0.320]. Again, main effects were found for emotion [*F*_(4,445)_ = 193.07, *p* < 0.001] and intensity [*F*_(3,93)_ = 92.90, *p* < 0.001]—with the highest scores for happiness and anger, and higher performance with increasing emotional intensity (Supplementary Fig. 1). *C9orf72* mutation carriers performed worse in recognizing anger at the lowest (40%) emotional intensity than controls (*p* = 0.038), and *GRN* mutation carriers (*p* = 0.047), but no other interaction effects were found between mutation carriers and controls [*F*_(38,1292)_ = 1.18, *p* = 0.22]. For happiness, group differences were larger at the lowest intensity (40%) than at the highest intensity (100%) (trend; *p* = 0.082), whereas for sadness, group differences showed an opposite pattern (*p* = 0.021).

### Classification abilities of the ERT

The classification abilities of the ERT total scores and emotion subscores can be found in Table 2. The ERT total score differentiated well between subgroups (*X*^2^(138) = 213.072, *p* < 0.001), with significant discriminative ability between patients with bvFTD and presymptomatic mutation carriers (*X*^2^(1) = 19.752, *p* < 0.001), patients with bvFTD and controls (*X*^2^(1) = 16.308, *p* < 0.001), patients with AD and presymptomatic mutation carriers (*X*^2^(1) = 22.325, *p* < 0.001), patients with AD and controls (*X*^2^(1) = 20.352, *p* < 0.001), but neither between patients with bvFTD and AD (*X*^2^(1) = 0.574, *p* = 0.449) nor between the presymptomatic mutation carriers and controls (*X*^2^(1) = 2.185, *p* = 0.139). A model consisting of the emotions anger, fear, happiness and surprise correctly classified 93.7% of patients with bvFTD and presymptomatic mutation carriers (*Χ*^2^(1) = 9.680, *p* = 0.002). The model with anger and happiness differentiated best (87.7% correctly classified) between patients with bvFTD and controls (*Χ*^2^(1) = 11.327, *p* = 0.001). The classification accuracy between patients with bvFTD and AD was low, just above chance level (59.4% correct), with only the emotion happiness being a significant predictor of the presenting phenotype (*Χ*^2^(1) = 5.368, *p* = 0.021). The ERT classified well (87.3% correctly classified) between patients with AD and presymptomatic mutation carriers with anger, disgust, and happiness as predictors (*Χ*^2^(1) = 13.211, *p* < 0.001). A similar model classified best (87.7% correct) between patients with AD and controls *Χ*^2^(1) = 16.155, *p* < 0.001). As can be expected from similar scores on the ERT, discriminative ability was low between presymptomatic mutation carriers and controls (64.6% correct), with only disgust being a significant classifier between groups (Table 2). Rerunning our analyses using centre as additional covariate did not change our results significantly.

**Table 2:** Classification abilities of the ERT per subgroup

	AUC	95% CI	*p* value	Optimal cut-off	Sensitivity (%)	Specificity (%)
bvFTD vs. AD						
Total score	0.52	0.34–0.63	0.830	–	–	–
Anger	0.55	0.40–0.67	0.532	–	–	–
Disgust	0.57	0.29–0.57	0.320	–	–	–
Fear	0.58	0.27–0.56	0.245	–	–	–
Happiness	0.63	0.49–0.77	0.086	–	–	–
Sadness	0.59	0.26–0.55	0.197	–	–	–
Surprise	0.51	0.34–0.63	0.856	–	–	–
bvFTD vs. presymptomatic carriers						
Total score	0.83	0.72–0.95	< 0.001	50.5	89.4	78.1
Anger	0.89	0.82–0.96	< 0.001	12.5	74.5	90.6
Disgust	0.81	0.71–0.91	< 0.001	9.5	85.1	68.8
Fear	0.54	0.32–0.60	0.566	–	–	–
Happiness	0.87	0.79–0.96	< 0.001	14.5	78.7	84.4
Sadness	0.59	0.45–0.73	0.176	–	–	–
Surprise	0.62	0.48–0.75	0.069	–	–	–
bvFTD vs. controls						
Total score	0.81	0.69–0.92	< 0.001	43.5	95.9	62.5
Anger	0.88	0.81–0.95	< 0.001	12.5	73.5	90.6
Disgust	0.72	0.61–0.84	< 0.001	7.5	77.6	59.4
Fear	0.54	0.41–0.68	0.517	–	–	–
Happiness	0.88	0.80–0.97	< 0.001	14.5	85.7	84.4
Sadness	0.57	0.43–0.72	0.277	–	–	–
Surprise	0.57	0.43–0.71	0.273	–	–	–
Presymptomatic carriers vs. controls						
Total score	0.59	0.48–0.71	0.111	–	–	–
Anger	0.52	0.40–0.63	0.764	–	–	–
Disgust	0.62	0.50–0.73	0.045	11.5	63.3	66.0
Fear	0.51	0.39–0.62	0.918	–	–	–
Happiness	0.50	0.39–0.62	0.947	–	–	–
Sadness	0.56	0.44–0.68	0.317	–	–	–
Surprise	0.58	0.47–0.70	0.174	–	–	–
AD vs. controls						
Total score	0.90	0.82–0.98	< 0.001	48.5	83.7	90.6
Anger	0.84	0.75–0.93	< 0.001	12.5	73.5	81.2
Disgust	0.82	0.73–0.91	< 0.001	9.5	61.2	90.6
Fear	0.57	0.45–0.70	0.284	–	–	–
Happiness	0.87	0.78–0.95	< 0.001	14.5	85.7	84.4
Sadness	0.73	0.61–0.84	0.001	4.5	65.3	71.9
Surprise	0.59	0.46–0.73	0.153	–	–	–

*ERT* Emotion Recognition Task, *AUC* area under the curve, *CI* confidence interval, *bvFTD* behavioural variant frontotemporal dementia, *AD* Alzheimer’s Dementia

## Discussion

This study is the first to examine emotion recognition abilities of dynamically morphed facial expressions in a large cohort of patients with bvFTD and AD, presymptomatic mutation carriers, and cognitively unimpaired control subjects, by means of the ERT. Across all emotions and intensities, patients with bvFTD and AD performed the worst, whereas highest scores were found in the total group of presymptomatic mutation carriers and controls, in which performance did not differ. Overall test performance was highest for anger and happiness, on which patients with bvFTD performed significantly worse than patients with AD. Presymptomatic *C9orf72* mutation carriers performed worse than presymptomatic *GRN* mutation carriers and controls on the 40% intensity level of the emotion disgust. The ERT classified well between patients with bvFTD and controls, patients with AD and controls, but could neither discriminate bvFTD from AD patients nor presymptomatic mutation carriers from controls. A model that included anger, fear, happiness and surprise correctly classified 93.7% of patients with bvFTD and presymptomatic mutation carriers.

Our finding that patients with bvFTD perform low across all emotions of the ERT is in line with a large number of studies showing significant impairments in emotion recognition in bvFTD [e.g., 10,14,30,31]. Neuroimaging studies have demonstrated a key role for the anterior temporal, orbitofrontal and insular cortex and a number of subcortical areas in emotional processing [32, 33], brain regions known to be heavily affected early in the disease process of bvFTD [34, 35]. Although there is general consensus that emotion recognition is impaired in bvFTD, the literature about the range to which (diffuse vs. selective) and the types of emotions (positive vs. negative) are affected shows mixed findings [36]. In line with, for instance, Keane et al. [37] and Kessels et al. [14], we found evidence for the presence of specific impairments in the recognition of anger, disgust and happiness in our bvFTD patient sample. Regarding the latter, contradicting findings have been found for positive emotions, with some studies showing preservation [e.g., 10,14,38,39] and others showing deficits [e.g., 37,40] in the identification of happy facial expressions. Regardless of relative higher performance in comparison to the other emotions, no ceiling effects for happiness were found in our study—an explanation brought forward by previous studies for the relative preservation of recognition of happiness [10]. We can infer from our findings that atrophy in bvFTD is likely not only specific to brain regions involved in negative emotions [10], but also affects brain regions involved in positive emotion processing, explaining global emotion recognition impairments in our bvFTD sample. This notion is in line with previous studies suggesting two different subtypes of bvFTD: a temporal variant with selective deficits in the recognition of negative emotions, and a frontal variant with both impairments in the recognition of negative *and* positive emotions [31, 37, 41]. One explanation for our findings is that our bvFTD patients had a predominant frontal or mixed frontotemporal pattern of atrophy—unfortunately MRI scanning was only performed in a subset of patients, and therefore, we could not include neuroimaging data in the present study.

Emotion recognition deficits were also found in our AD group, wherein patients scored lower than presymptomatic mutation carriers and controls on the ERT total score and lower disgust scores than presymptomatic mutation carriers, resulting in overall good classification accuracy between the two groups. These findings are consistent with previous studies demonstrating significant emotion recognition impairments in patients with AD [15, 42, 43], thereby contrasting the notion that impairment of emotion recognition is relatively unique for the frontotemporal dementia spectrum [10]. As the brain areas involved in emotion recognition also tend to be affected in patients with AD [41], this is not a surprising finding. It might explain that, although patients with bvFTD performed worse on the emotions anger and happiness than patients with AD, the differences were smaller than previously reported [10]. Another potential explanation can be found in the commonly atypical presentations of patients with AD, we see in our outpatient memory clinic, such as ‘frontal’ (dysexecutive and behavioural) variants, in which there is potentially more clinical overlap with bvFTD. As most clinical diagnoses were not pathologically confirmed (e.g., using AD biomarkers in CSF), the small possibility remains that patients with frontal AD presentations have been diagnosed as bvFTD, and bvFTD patients with prominent memory deficits as patients with AD, thereby decreasing classification accuracy between the two groups in our study.

The presymptomatic mutation carrier group as a whole did not differ significantly from cognitively unimpaired controls on the ERT total score and emotion subscores. Prior research in presymptomatic familial FTD so far has been scarce, with only a few studies investigating social cognition in *MAPT* [11, 44, 45] and *GRN* [11, 44] mutation carriers. In our previous study in the FTD-RisC cohort, we demonstrated longitudinal presymptomatic decline in emotion recognition (by means of the Ekman Faces test) in *MAPT* mutation carriers and in theory of mind (by means of the Happé cartoons test) in *GRN* mutation carriers [11]. Direct comparison to this study is—however—complicated, as different statistical methods (e.g., a cross-sectional approach in this study vs. longitudinal modelling, and using estimated years to symptom onset (EYO) in the previous study) and instruments were used. The same goes for the study by Cheran et al. [45], in which mostly observer-based measures of social cognition were employed. As a next step, it will be interesting to explore the potential of the ERT in mutation carriers closer to overt disease (‘converters’) [46] than the presymptomatic mutation carriers investigated in this study, allowing us to further explore emotion recognition deficits in early-stage FTD. Our study is the first to demonstrate emotion recognition deficits at the lowest emotional intensity in presymptomatic *C9orf72* mutation carriers. It could be hypothesized that this is related to early changes in socio-emotional cognition linked to the selective vulnerability and loss of von Economo neurons, which is specifically characteristic of bvFTD due to *C9orf72* [47]. We did not find differences on the ERT between bvFTD patients carrying the *C9orf72* mutation and sporadic or concomitant ALS bvFTD patients. This is in line with previous research, demonstrating that—although there can be some clinical heterogeneity—the cognitive profiles between, respectively, *C9orf72*-bvFTD and sporadic bvFTD [48, 49] and between sporadic bvFTD and FTD-ALS [50] are remarkably similar. This strengthens our idea of bvFTD as a disease spectrum, though with common deficits in social cognition.

We find large differences in emotion subscores regardless of clinical status, with relatively high scores for anger and happiness, low scores for fear, intermediate scores for surprise, and more variable scores for disgust and sadness. The overall high anger and happiness scores, and low fear scores are consistent with the results from Kessels et al. [14], and are most likely the result of task difficulty (i.e. the recognition of fearful expressions is regarded as difficult, even by cognitively unimpaired controls) [13], whereas variability in subscores could be related to the ambiguity of some items (i.e. happiness and anger are more uniformly portrayed than disgust and surprise, specifically at lower intensities). Near-ceiling performances were found for happiness above 60% intensity in presymptomatic mutation carriers and controls. This preservation could stem from the statistical artefact of only having one positive emotion to choose from when using the six basic emotions, whereas the recognition of negative emotions is more difficult as one has more answer choices (e.g., fear, sadness, anger, disgust) [36]. In contrast to studies finding ceiling effects in bvFTD using static emotions [10, 30], use of the ERT which includes presentation of emotional morphs at lower intensities, results in small deficits in the presymptomatic stage of *C9orf72*-FTD, underlining the importance of using more sensitive cognitive tasks to improve early diagnosis. This is further corroborated by our findings of increasing task performance with higher emotional intensity, and better discrimination between groups at the lowest than at the highest emotional intensity, where the latter condition resembles the full-blown intensity used in static paradigms.

Key strengths of our study constitute our large groups of presymptomatic mutation carriers from *MAPT*, *GRN* and *C9orf72* families, patients with bvFTD and AD, and controls. Although the ERT has been investigated in a small convenience sample of bvFTD [14], this study is the first to make the direct comparison between patients with AD and bvFTD, and to investigate the presymptomatic phase of FTD. Our results should be replicated in our own longitudinal as well as larger international cohorts, such as GENFI [51], allowing us to draw firmer conclusions with respect to emotion recognition deficits in early-stage FTD. The use of patient cohorts from three different centres may have potentially introduced some heterogeneity into our patient samples, although rerunning our analyses using centre as additional covariate did not change our results significantly. Directions for future research entail increasing and expanding group samples, and including MCI-AD and PPA patients. Moreover, investigating neuroimaging as well as cognitive correlates could increase our insight into the erosion of neural networks thought to underlie behavioural and emotional changes in early-stage FTD. Lastly, it would be interesting to explore a fuller range of emotions than the basic six investigated here, for instance self-conscious emotions (e.g., embarrassment, shame, guilt, contempt) that are thought to be particularly important for effective social functioning [36], and to investigate more modalities than visual perception alone along higher hierarchical levels of social cognition to get a full understanding of changes in conversion from presymptomatic to symptomatic stages of FTD.

### Conclusion

Our study demonstrates the presence of emotion recognition deficits of morphed facial expressions by means of the ERT in patients with bvFTD and AD, but not in cognitively unimpaired controls or presymptomatic FTD mutation carriers, apart from minor deficits in recognizing anger at the lowest emotional intensity in *C9orf72* mutation carriers. The ERT classified well between patients with bvFTD and controls/presymptomatic mutation carriers, patients with AD and controls/presymptomatic mutation carriers, but not between patients with bvFTD and AD nor presymptomatic mutation carriers and controls. Our results demonstrate clear emotion recognition deficits in bvFTD and AD patients, and points towards the presence of subtle changes in facial emotion recognition in presymptomatic FTD due to the *C9orf72* mutation. This highlights the importance of incorporating dynamic emotion recognition paradigms such as the ERT into the standard neuropsychological assessment for early differential diagnosis in dementia and as potential clinical endpoints in upcoming therapeutic trials for FTD and AD.

## Electronic supplementary material

Below is the link to the electronic supplementary material.

Supplementary Fig. 1. Mean performance (y-axis, number correctly identified emotions = max 4) of presymptomatic FTD mutation carriers with an MAPT mutation (light blue), GRN mutation (light green), or C9orf72 repeat expansion (dark blue) and cognitively unimpaired controls (grey) for the six different emotions across the emotional intensities (x-axis). (TIF 8903 kb)

Supplementary file2 (DOCX 16 kb)
