# Supplementary figures and images for: Emotion recognition of morphed facial expressions in presymptomatic and symptomatic frontotemporal dementia, and Alzheimer’s dementia
Source: J Neurol. 2020 Jul 29;268(1):102–13. doi: 10.1007/s00415-020-10096-y (PMC7815624; doi:10.1007/s00415-020-10096-y)

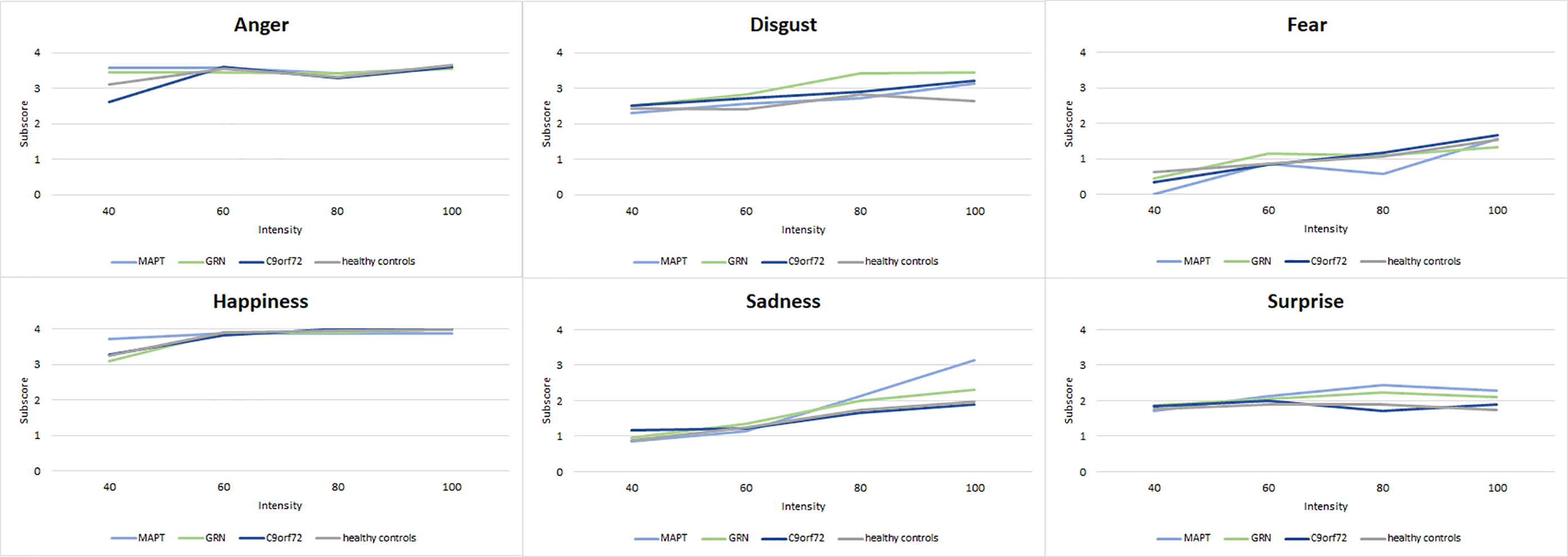

Supplement: Supplementary file 1 — Supplementary Fig. 1. Mean performance (y-axis, number correctly identified emotions = max 4) of presymptomatic FTD mutation carriers with an MAPT mutation (light blue), GRN mutation (light green), or C9orf72 repeat expansion (dark blue) and cognitively unimpaired controls (grey) for the six different emotions across the emotional intensities (x-axis). (TIF 8903 kb) [file 415_2020_10096_MOESM1_ESM.tif]
