# Supplementary material for: Emotion recognition of morphed facial expressions in presymptomatic and symptomatic frontotemporal dementia, and Alzheimer’s dementia
Source: J Neurol. 2020 Jul 29;268(1):102–13. doi: 10.1007/s00415-020-10096-y (PMC7815624; doi:10.1007/s00415-020-10096-y)
Supplement: Supplementary file 2 — Supplementary file2 (DOCX 16 kb) [file 415_2020_10096_MOESM2_ESM.docx]

Supplementary Table 1. ERT total and subscores of sporadic bvFTD patients, bvFTD patients carrying the *C9orf72* mutation, and bvFTD patients with concomitant ALS.

|  | Sporadic bvFTD (n=21) | *C9orf72*-bvFTD (n=6) | bvFTD with concomitant ALS (n=5) | Test statistics | |
| --- | --- | --- | --- | --- | --- |
| Total score | 36.9 ± 7.9 | 38.5 ± 10.2 | 31.6 ± 3.6 | F(2,0.588) = 3.14 | p=0.567 |
| Anger | 8.3 ± 3.6 | 7.7 ± 6.0 | 8.6 ± 3.0 | H(2) = 0.018 | p=0.991 |
| Disgust | 7.8 ± 4.5 | 6.2 ± 2.7 | 3.8 ± 4.4 | H(2) = 3.832 | p=0.147 |
| Fear | 7.4 ± 6.0 | 2.5 ± 1.6 | 2.0 ± 1.0 | H(2) = 4.269 | p=0.118 |
| Happiness | 8.2 ± 6.1 | 12.8 ± 1.3 | 11.2 ± 4.6 | H(2) = 1.901 | p=0.386 |
| Sadness | 7.0 ± 4.8 | 3.3 ± 2.3 | 2.4 ± 2.5 | H(2) = 4.878 | p=0.087 |
| Surprise | 8.1 ± 4.3 | 6.0 ± 2.9 | 3.6 ± 2.2 | H(2) = 5.752 | p=0.056 |

Values indicate: mean ± standard deviation. Abbreviations: ERT, Emotion Recognition Test; bvFTD, behavioural variant frontotemporal dementia; *C9orf72*, Chromosome 9 open reading frame 72; ALS, amyotrophic lateral sclerosis. Data were analysed using one-way analysis of variance (ANOVA) for normally distributed data (F-statistic), or Kruskal-Wallis tests for non-normally (nonparametric) data (H-statistic).
